# Restoration of amyloid PET images obtained with short-time data using a generative adversarial networks framework

**DOI:** 10.1038/s41598-021-84358-8

**Published:** 2021-03-01

**Authors:** Young Jin Jeong, Hyoung Suk Park, Ji Eun Jeong, Hyun Jin Yoon, Kiwan Jeon, Kook Cho, Do-Young Kang

**Affiliations:** 1 Department of Nuclear Medicine, Dong-A University Hospital, Dong-A University College of Medicine, 1, 3ga, Dongdaesin-dong, Seo-gu, Busan, 602-715 South Korea; 2 Institute of Convergence Bio-Health, Dong-A University, Busan, Republic of Korea; 3 Department of Translational Biomedical Sciences, Dong-A University, Busan, Republic of Korea; 4 National Institute for Mathematical Science, Daejeon, Republic of Korea; 5 College of General Education, Dong-A University, Busan, Republic of Korea

**Keywords:** Medical research, Molecular medicine, Neurology, Mathematics and computing

## Abstract

Our purpose in this study is to evaluate the clinical feasibility of deep-learning techniques for F-18 florbetaben (FBB) positron emission tomography (PET) image reconstruction using data acquired in a short time. We reconstructed raw FBB PET data of 294 patients acquired for 20 and 2 min into standard-time scanning PET (PET_20m_) and short-time scanning PET (PET_2m_) images. We generated a standard-time scanning PET-like image (sPET_20m_) from a PET_2m_ image using a deep-learning network. We did qualitative and quantitative analyses to assess whether the sPET_20m_ images were available for clinical applications. In our internal validation, sPET_20m_ images showed substantial improvement on all quality metrics compared with the PET_2m_ images. There was a small mean difference between the standardized uptake value ratios of sPET_20m_ and PET_20m_ images. A Turing test showed that the physician could not distinguish well between generated PET images and real PET images. Three nuclear medicine physicians could interpret the generated PET image and showed high accuracy and agreement. We obtained similar quantitative results by means of temporal and external validations. We can generate interpretable PET images from low-quality PET images because of the short scanning time using deep-learning techniques. Although more clinical validation is needed, we confirmed the possibility that short-scanning protocols with a deep-learning technique can be used for clinical applications.

## Introduction

Amyloid positron emission tomography (PET) is a nuclear medicine imaging test that shows amyloid deposits in the brain. It is currently being used in the diagnosis of Alzheimer's disease, which is known to be caused by amyloid^1^. Although there are some differences in the acquisition protocols that depend on the commercially available radiopharmaceuticals for amyloid PET, most of these should be taken for 10–20 min, especially for F-18 florbetaben (FBB), which needs 20 min for scanning^2^. Since most of the patients with memory disorder are elderly, there are complaints that it is difficult for them to lie down without movement for 20 min. Head movements due to postural discomfort during long scan acquisition can cause motion artifacts in PET images, which degrade their diagnostic value. Some elderly patients actually needed re-scanning (or additional radiation exposure) because of a poor image due to movement. Thus, the demand for shortening scan time is growing with the increasing use of PET for patients with dementia. However, PET images obtained from short scanning times can suffer from a low signal-to-noise ratio and have reduced diagnostic reliability as well.

Recently, deep-learning techniques for image restoration have been widely applied to medical images, including computed tomography (CT), magnetic resonance imaging (MRI), and PET^3–11^. Some of them have used the deep-learning techniques for low-dose PET image restoration and have shown potential for reducing noise artifacts^3–8^. There have been only a few studies on reducing noise and improving the quality of images taken by reducing the acquisition time of brain PET^7^. They have used additional MR information obtained from a PET/MR scanner to restore brain PET images. However, a PET/MR scanner is costly and is not yet widely installed. Since PET/CT scanners are used in most hospitals, a restoration technique using only PET without MRI information is needed.

In this study, we applied the deep-learning technique for short-scanning FBB PET image restoration. The proposed method uses PET images only, without additional information, such as MRI or CT. We did qualitative and quantitative analyses to evaluate the clinical applicability of the proposed method.

## Materials and methods

The Institutional Review Board (IRB) of Dong-A University Hospital reviewed and approved this retrospective study protocol (DAUHIRB-17-108). The IRB waived the need for informed consent, since only anonymized data would be used for research purposes. We used all methods in accordance with the relevant guidelines and regulations.

### Patients and F-18 FBB brain PET acquisition

For training and internal validation of our deep-learning algorithm, we enrolled 294 patients with clinically diagnosed cognitive impairment who had received FBB PET between December 2015 and May 2018 retrospectively in this study. We also randomly collected 30 patients who had FBB PET from January to May 2020 for temporal validation. Out of 30 patients, we excluded two patients because of insufficient clinical information, and finally 28 patients participated. In this study, we excluded patients with head movement during PET scanning. All the FBB PET examinations were done using a Biograph mCT flow scanner (Siemens Healthcare, Knoxville, TN, USA). The PET/CT imaging was done according to the routine examination protocol of our hospital, which is the same method used in the previous study published by our group^12^. We injected 300 MBq F-18 florbetaben intravenously into the patients and started PET/CT acquisition 90 min after the radiotracer injection. A helical CT scan was carried out with a rotation time of 0.5 s at 120 kVp and 100 mAs, without an intravenous contrast agent. A PET scan followed immediately, and the image was acquired for 20 min with the list mode. All the images were acquired from the skull vertex to the skull base. We reconstructed the list mode PET data for 20 min into a 20-min static image (PET_20m_) and used it as the full-time ground-truth image. We also reconstructed a short-scanning static PET image (PET_2m_) using the first 2-min data from the total list mode PET data. We used the same parameters to acquire both PET_20m_ and PET_2m_ images.

In addition, we carried out external validation, and obtained data used in the preparation of the external validation from the Alzheimer’s Disease Neuroimaging Initiative (ADNI) database (http://adni.loni.usc.edu). Among the subjects who underwent FBB PET, we randomly selected 60 patients, and excluded two patients because of inconsistency in the brain amyloid plaque load (BAPL) scoring. Finally, 58 patients were involved.

The characteristics of all subjects included in this study are summarized in Table 1.

**Table 1:** Subjects’ characteristics.

Parameters	Training set	Internal validation set	Temporal validation set	External validation set
Number of subjects (n)	236	58	28	58
Woman	143	33	18	31
Man	93	25	10	27
**Age (years)**				
Mean	69.8 ± 7.4	71.6 ± 7.5	69.8 ± 8.3	71.4 ± 7.3
Range	52–86	51–84	54–84	56–89
**MMSE score**				
Mean	22.9 ± 4.8	23.4 ± 4.8	18.5 ± 6.3	27.8 ± 3.0
Range	9–30	10–30	5–29	13–30
**Clinical diagnosis (n)**				
Normal	15	5	5	21
SCD	35	10	1	0
MCI	70	16	6	31
AD	116	27	16	6
**BAPL score (n)**				
1/2/3	112/25/99	16/18/24	13/2/13	25/12/21

Values of age and MMSE score are presented as mean ± SD (standard deviation).

*AD* Alzheimer’s disease, *BAPL* brain amyloid plaque load, *MMSE* mini-mental state examination.

### Deep-learning method

#### Network architecture

**Figure 1:**
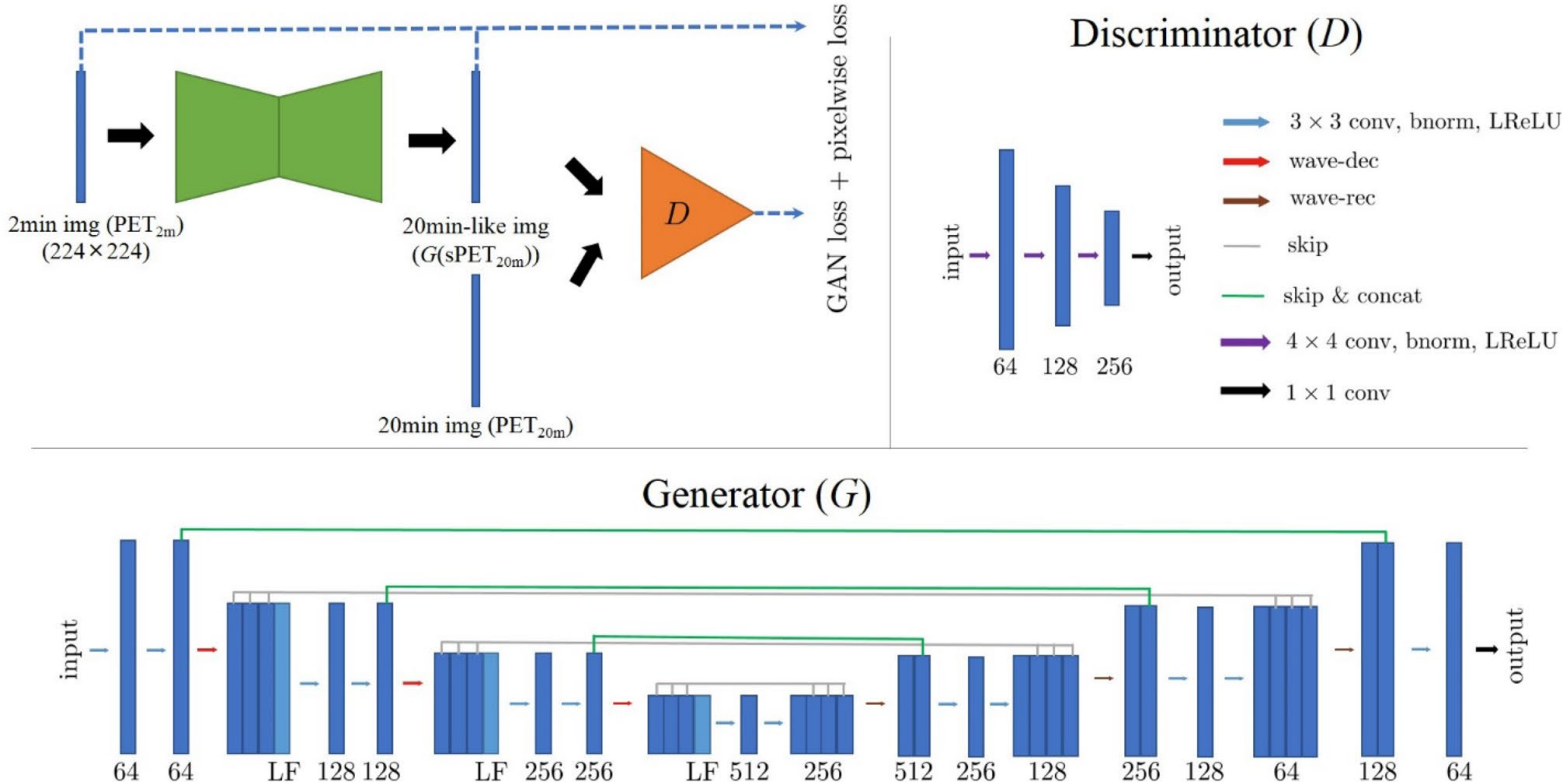
The schematic diagram of the adversarial network used in this study (top left). In this proposed network, the discriminator (top right) and the generator (bottom row) are shown, and the generator is constructed using the deep convolutional framelets. The numbers below the rectangular boxes indicate the number of filters.

We adopted a generative adversarial network that consists of two competing neural networks with an additional pixelwise loss^13^. The schematic diagram of the proposed network is shown in Fig. 1. The generator ($G$) is trained to generate a synthetic PET_20m_-like (sPET_20m_) image from the noisy PET_2m_ image, and the discriminator ($D$) is trained to distinguish sPET_20m_ images generated by the generator from real PET_20m_ image. In the training procedure, the discriminator enables the generator to provide more realistic sPET_20m_ images^14^. Pixelwise loss is defined as a mean-squared error between sPET_20m_ images and original PET_2m_ images, which prevents the generator from changing small anomalies or structures of PET_2m_ images during training^15^.

The generator is constructed using the deep convolutional framelets, which consist of encoder-decoder structures with skipped connections^16^. Both encoder and decoder paths contain two repeated $3\times 3$ convolutions (conv), each followed by a batch normalization (bnorm) and a leaky rectified linear unit (LReLU)^17, 18^. A 2-D Haar wavelet de-composition (wave-dec) and re-composition (wave-rec) are used for down-sampling and up-sampling, respectively, of the features^19^. In the encoder path, three high-pass filters after wavelet de-composition skip directly to the decoder path (arrow marked by ‘skip’), and one low-pass filter (marked by ‘LF’) is concatenated with the features in the encoder path at the same step (arrow marked by ‘skip & concat’). At the end, a convolution layer with a $1\times 1$ window is added to match the dimension of input and output images. The numbers below the rectangular boxes in Fig. 1 indicate the number of filters. The architecture of deep convolutional framelets is similar to that of the U-net^20^, a standard multi-scale convolutional neural network (CNN) with skipped connections. The difference is in using the wavelet de-composition and re-composition, instead of max-pooling and un-pooling, for down-sampling and up-sampling, respectively. Additional skip connections of high-frequency filters help to train the detailed relationship between PET_2m_ and PET_20m_ images.

For the discriminator, we adopted the standard CNN without a fully connected layer. The discriminator contains three convolution layers with a $4\times 4$ window and strides of two in each direction of the domain, each followed by a batch normalization and a leaky ReLU with a slope of 0.2. At the end of the architecture, a $1\times 1$ convolution is added to generate a single-channel image.

#### Datasets for training and internal validation

In the dataset of the 294 patients’ PET images (70 image slices/patient), we randomly divided the training and internal validation datasets into 80% and 20%, and used 236 patients’ images as the training dataset and used 58 patients’ images as the internal validation dataset. The original size of the PET images was $400\times 400$. In order to improve training effectiveness, we cropped all $400\times 400$ images to $224\times 224$ pixels around the center of an image in both horizontal and vertical directions. Here, only background (i.e., zero-valued) information was removed. We used the cropped images as input and label datasets for the proposed deep-learning network. In the testing procedure, we resized the images corrected by means of the trained generator to $400\times 400$ by adding the rows and columns of zeros at the top, bottom, left, and right sides of the images (i.e., zero padding). We did not use data augmentations such as rotation or flipping for training.

#### Network training

In our study, we ran training for 200 epochs using Adam solver with a learning rate of 0.0002, and a mini-batch size of 10^21^. It was implemented using TensorFlow on a CPU (Intel Core i9-7900X, 3.30 GHz) and a GPU (NVIDIA, Titan Xp. 12 GB) system^22^. It took about 68 h to train the network. The network weights followed a Gaussian distribution, with a mean of 0 and a standard deviation of 0.01.

### Assessment of image quality

We compared the image quality of PET_2m_ and the synthesized sPET_20m_ images with the original PET_20m_ images using the peak signal-to-noise ratio (PSNR), structural similarity (SSIM), and normalized root mean-square error (NRMSE). The SSIM index depends on the parameters ${C}_{1}= {({K}_{1}L)}^{2}$ and ${C}_{2 }= {({K}_{2}L)}^{2}$, where $L$ is the dynamic range of pixel values and $K$ is some constant^8^. In our study, we chose ${C}_{1} = {\left(0.0002\times 65535\right)}^{2}\mathrm{ and }{C}_{2} = {\left(0.0007\times 65535\right)}^{2}$. The proposed method was also compared with the standard U-net method.

For further analysis, we calculated the Standardized Uptake Value Ratio (SUVR) using PMOD 3.6 software (PMOD Technologies, Zurich, Switzerland)^23^. We obtained the transformation matrix of each participant by fusing the CT template of the PMOD and the CT image of the participant. PET images were then spatially normalized by using the transformation matrix of each participant and were applied to an automated anatomical labeling template of PMOD (Hammers atlas). We spatially normalized all pairs of sPET_20m_ and PET_20m_ images to the Montreal Neurological Institute (MNI) spatial templates and applied the Hammers atlas. By reconstructing the volume-of-interests of the atlas, the representative areas were set up as the striatum, frontal, parietal, temporal and occipital lobes, and global brain. We calculated the SUVRs of the representative areas and used the cerebellar cortex as the reference region. We compared the difference of SUVRs of the identical area between sPET_20m_ and PET_20m_ images.

### Clinical interpretations

For visual interpretation, three nuclear medicine physicians with certification and experience in amyloid PET readings participated (YJ and DY have over 15 years and JE has 4 years of experience in nuclear medicine; all of them also have 4 years of experience in amyloid PET assessment). They were blinded to the clinical data and independently read all PET images of the internal validation dataset.

#### Turing test

We did two Turing tests and evaluated all PET images of the internal validation dataset. First, of all the sPET_20m_ and PET_20m_ images, we randomly selected 58 images and presented them to the physicians one by one for them to decide whether the PET image was real or synthetic (Test 1). Second, we presented a pair of sPET_20m_ and PET_20m_ images of the same patient to the physicians to find the original PET_20m_ image (Test 2). We anonymized all PET images and randomized the order of PET images.

#### BAPL score

We gave all anonymized sPET_20m_ images of the internal validation dataset to the physicians to interpret and score according to the conventional interpretation protocol. All the sPET_20m_ images were classified into three groups according to the BAPL scoring system. BAPL score is a specialized, predefined three-grade scoring system for F-18 FBB PET wherein measurements are made by the physician based on the visual assessment of the subject’s amyloid deposits in the brain^24^. BAPL scores of 1 (BAPL 1), 2 (BAPL 2), and 3 (BAPL 3) indicate no amyloid load, minor amyloid load, and significant amyloid load, respectively. Therefore, BAPL 1 indicates a negative amyloid deposit, whereas BAPL 2 and BAPL 3 represent positive amyloid deposits. In this study, we treated the BAPL score read from the PET_20m_ images as the ground-truth score set by consensus among the three physicians. We measured the accuracy of the BAPL score for each physician. We also analyzed the agreements between the BAPL score of sPET_20m_ and PET_20m_ images for each physician.

### Temporal and external validations

We additionally verified our model by measuring PSNR, SSIM, NRMSE, and SUVR by means of temporal and external validation. The patient characteristics of our temporal and external validation datasets are illustrated in Table 1. We performed all analyses of temporal validation in the same manner as used in internal validation. We did external validation using a public FBB dataset from ADNI. ADNI datasets contain a series of 4 $\times$ 5 min of FBB PET images. The proposed model trained on our institute dataset (i.e., pair of 2-min and 20-min images) was tested on the first 5-min PET images. In this study, a Gaussian filter with 4-mm FWHM was applied to all FBB PET images of ADNI datasets.

### Statistical analysis

We assessed the intra-observer agreement of the BAPL score between the sPET_20m_ and PET_20m_ images using Cohen’s weighted kappa. We calculated the accuracy, sensitivity, and specificity for the interpretations of sPET_20m_ images. We assessed the difference of group characteristics using an independent *t*-test, one-way ANOVA, and chi-squared test. We evaluated the difference in SUVR between sPET_20m_ and PET_20m_ images using an independent *t*-test or Mann–Whitney U test, and evaluated the relationship of SUVRs between them using the Pearson’s correlation coefficient. We assessed agreement of SUVRs of both PET images using the Bland–Altman 95% limits of agreement. We did the statistical analyses using the MedCalc software version 16.4 (MedCalc Software, Mariakerke, Belgium) and NCSS 12 Statistical Software (NCSS, LLC. Kaysville, Utah, USA). Statistical significance was defined as *p* < 0.05.

## Results

### Assessment of image quality

#### PSNR, SSIM and NRMSE

**Figure 2:**
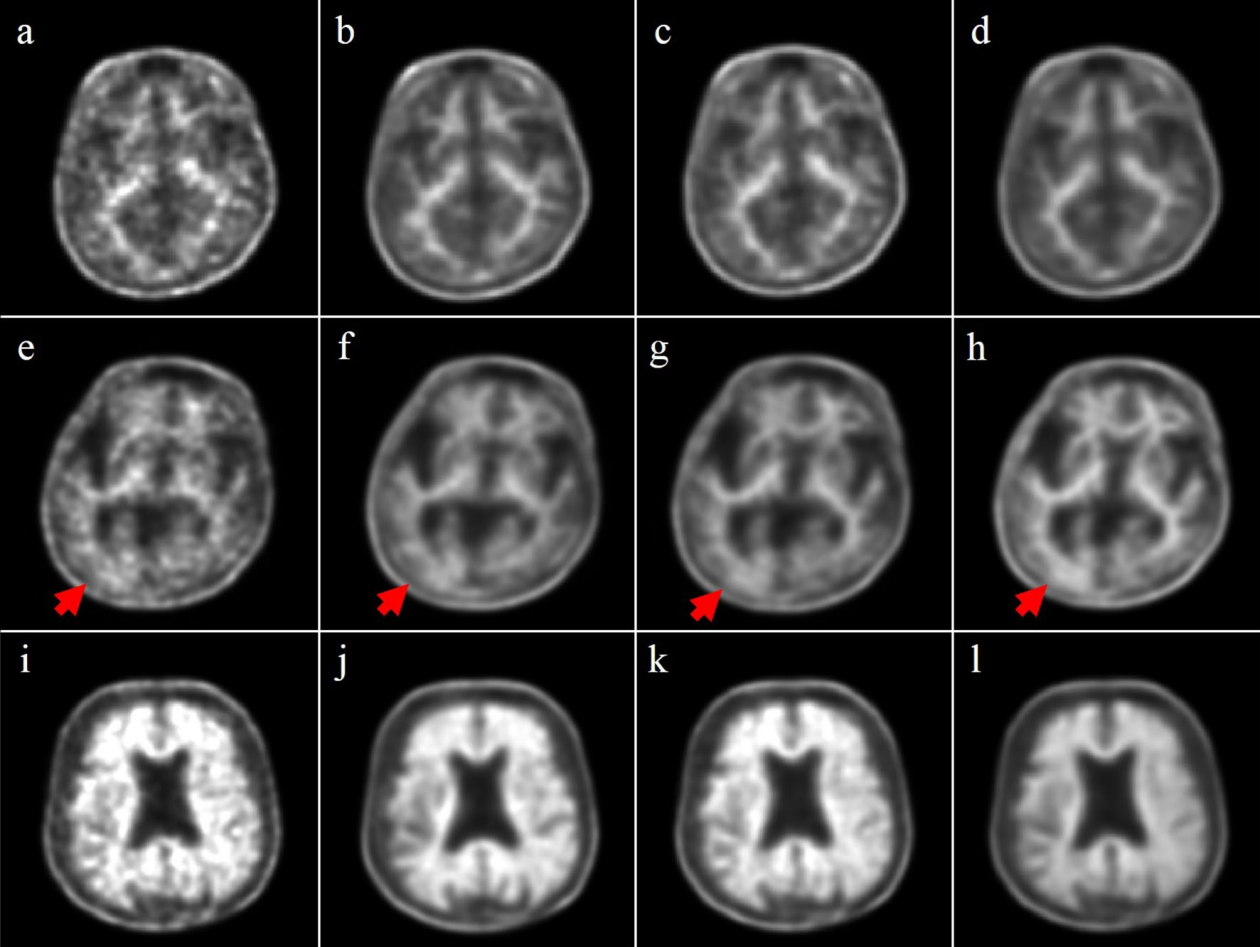
The input and output of PET images (upper row, BAPL 1; middle row, BAPL 2; lower row, BAPL 3). PET_2m_ image (input image) is very noisy and the image quality is poor (**a**,**e**,**i**). The ground truth with 20-min scanning (**b**,**f**,**j**) and synthetic PET images generated from the proposed deep learning (**c**,**g**,**k**) and the U-net (**d**,**h**,**l**) are shown. The synthetic PET image generated from our model is better in reflecting the underlying anatomical details than is the PET image generated from the U-net. In the BAPL 2 case, a small positive lesion (red arrows, **e**–**h**) is equivocal in the PET_2m_ image (**e**), but clearly shown in sPET_20m_ image (**g**) as in PET_20m_ image (**f**).

The original PET_2m_, PET_20m_ and sPET_20m_ images and a synthetic image generated by U-net are shown in Fig. 2.

Both the proposed and the U-net methods significantly reduce noise, but the U-net produces a slightly blurrier image than does the proposed method. For quantitative comparison, we calculated averaged PSNR, SSIM, and NRMSE for all datasets. The results are summarized in Table 2, which shows that the proposed method had the highest PSNR and SSIM, and lowest NRMSE, whereas PET_2m_ images showed the worst performance in internal validation. The proposed model shows similar performance for the temporal validation dataset, in terms of PSNR, SSIM, and NRMSE. As shown in Table 2, our method also improved the image qualities of the 5-min images in external validation.

**Table 2:** Image-quality metrics of PET image categories for internal, temporal, and external validation.

Metrics	Internal validation			Temporal validation		External validation	
	PET_2m_	sPET_20m_	U-net	PET_2m_	sPET_20m_	PET_5m_	sPET_20m_
PSNR	33.035	35.826	34.600	32.667	34.604	36.833	37.993
SSIM	0.844	0.882	0.869	0.848	0.873	0.862	0.871
NRMSE	15.421	11.286	12.912	15.992	12.992	12.097	11.004

*NRMSE* normalized root mean-square error, *PSNR* peak signal-to-noise ratio, *SSIM* structural similarity.

#### SUVR

##### Internal validation dataset

**Figure 3:**
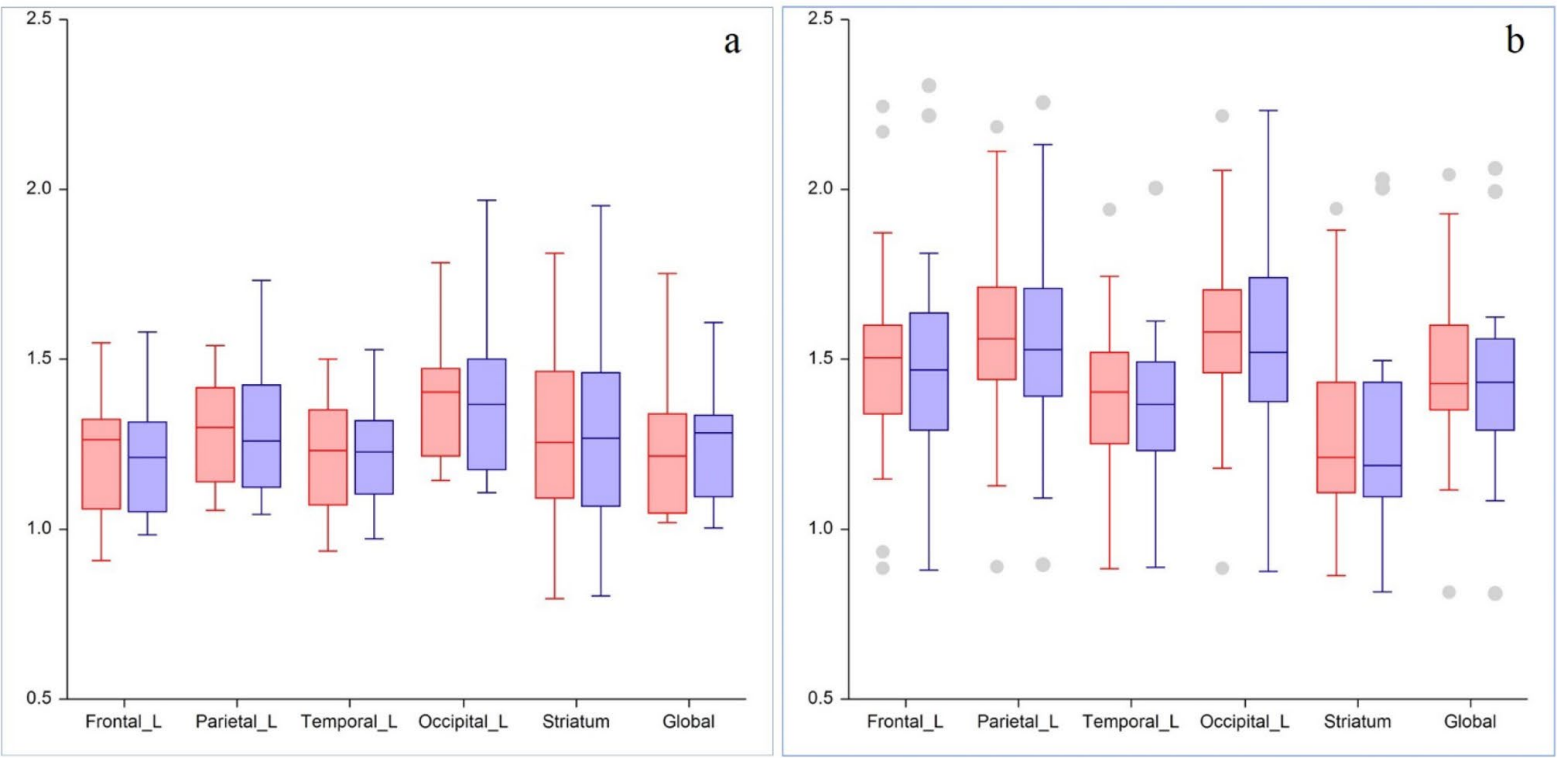
Comparison of the regional mean SUVR in the PET_20m_ (light blue) and sPET_20m_ (light red) images of the internal validation dataset (**a**, negative group; **b**, positive group). Similar values are shown between PET_20m_ and sPET_20m_ images.

**Figure 4:**
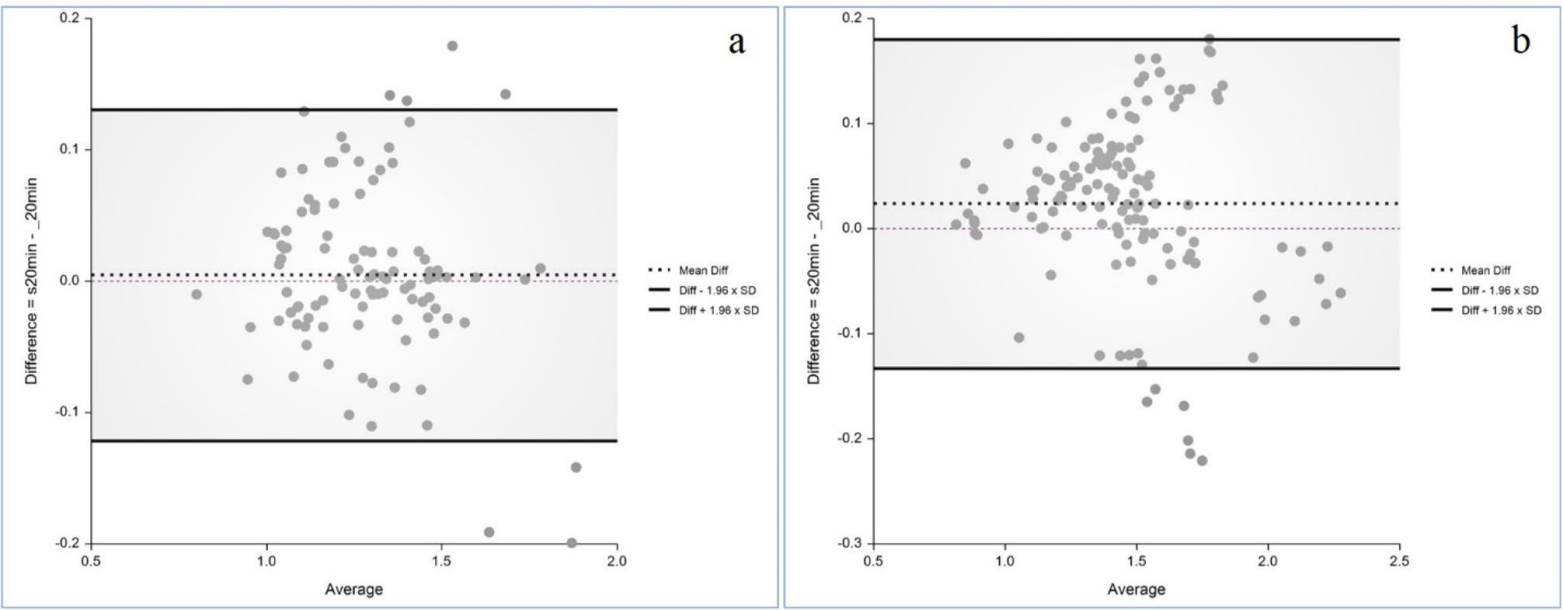
The Bland–Altman analysis for every regional SUVR shows a small mean difference between the PET_20m_ and sPET_20m_ images (**a**, negative group; **b**, positive group).

In internal validation, there was no statistically significant difference of SUVR between the PET_20m_ and sPET_20m_ images in the striatum, frontal, parietal, temporal, occipital lobes, and global brain (Fig. 3, Supplementary Table 1 and Fig. 1). In the Bland–Altman analysis, the regional mean difference of SUVR between sPET_20m_ and PET_20m_ images was 0.005 (95% confidence interval (CI) − 0.008, 0.017) in the negative group (Fig. 4a) and 0.024 (95% CI 0.010, 0.037) in the positive group (Fig. 4b). Upper and lower limits of agreement were 0.131 (95% CI 0.110, 0.152) and − 0.121 (95% CI − 0.142, − 0.100) in the negative group, and 0.180 (95% CI 0.157, 0.203) and − 0.133 (95% CI − 0.156, − 0.110) in the positive group, respectively.

##### Temporal and external validation datasets

**Figure 5:**
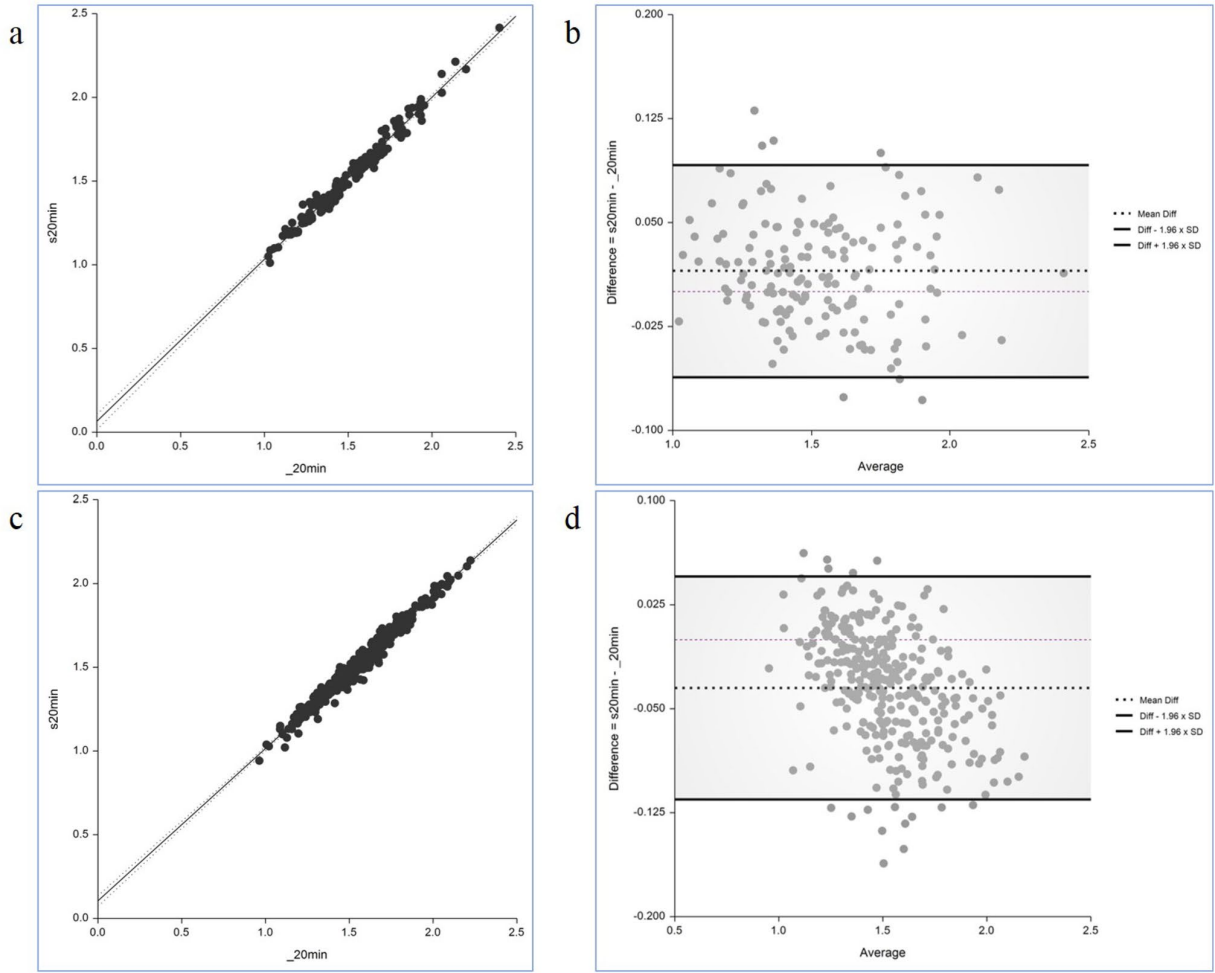
Correlation analysis of the whole representative areas shows a trend of a strong positive relationship of SUVR in temporal (**a**) and external validations (**c**). The Bland–Altman analysis shows a minimal mean difference between the two images in temporal (**b**) and external validations (**d**).

In temporal and external validations, we also compared SUVRs of the entire representative areas between sPET_20m_ and PET_20m_ images and found result similar to those of internal validation (Supplementary Tables 2, 3 and Figs. 2, 3). There was a very strong positive correlation between SUVRs of sPET_20m_ and PET_20m_ images in temporal validation (*r* = 0.988, *p* < 0.001, Fig. 5a) and external validation (*r* = 0.987, *p* < 0.001, Fig. 5c). In the Bland–Altman analysis, the mean difference of SUVR between sPET_20m_ and PET_20m_ images was 0.015 (95% CI 0.009, 0.021) in temporal validation (Fig. 5b). Upper and lower limits of agreement were 0.092 (95% CI 0.081, 0.102) and − 0.062 (95% CI − 0.072, − 0.051). In external validation, the mean difference of SUVR was − 0.035 (95% CI − 0.039, − 0.030) (Fig. 5d). Upper and lower limits of agreement were 0.045 (95% CI 0.038, 0.053) and − 0.115 (95% CI − 0.123, − 0.107).

### Clinical interpretations for internal validation dataset

#### Turing test

Tests 1 and 2 showed similar results (Table 3). Test 1, a test to decide whether the presented single PET image was real or synthetic, showed that, regardless of the duration of clinical reading experience in nuclear medicine, the overall accuracy was not high (44.8% and 63.8%). In Test 2, a test to select a real PET image out of two PET images of the same patient, the more experienced the physicians were in clinical reading, the more often the real PET image was selected (48.3–60.3%). Overall, however, the clinicians did not seem to distinguish well between generated PET images and real PET images.

**Table 3:** Accuracy of three physicians in two Turing tests.

Readers	Test 1	Test 2
4 years experienced physician	26/58 (44.8%)	28/58 (48.3%)
Over 15 years experienced physician 1	37/58 (63.8%)	35/58 (60.3%)
Over 15 years experienced physician 2	26/58 (44.8%)	32/58 (54.2%)

#### BAPL score

The three physicians assessed the sPET_20m_ images according to the BAPL scoring system, and there was no poor or inadequate image that was difficult to interpret. In five, six, and eight patients out of 58 patients, each physician assessed the BAPL score differently from the ground-truth score. Table 4 shows the accuracy, sensitivity, and specificity for the three physicians. Overall, the mean values for accuracy, sensitivity, and specificity were 89.1%, 91.3%, and 83.3%, respectively. The confusion matrices are provided in Table 5.

**Table 4:** Accuracy, sensitivity, and specificity in clinical reading using the BAPL score.

Metric	Reader 1	Reader 2	Reader 3	Mean (%)
Accuracy	91.4% (81.0, 97.1)	89.7% (78.8, 96.1)	86.2% (74.6, 93.9)	89.1
Sensitivity	95.2% (83.8, 99.4)	88.1% (74.4, 96.0)	90.5% (77.4, 97.3)	91.3
Specificity	81.3% (54.4, 96.0)	93.8% (69.8, 99.8)	75.0% (47.6, 92.7)	83.3

Data in parentheses are 95% confidence interval (%).

**Table 5:** Confusion metrics for interpretation of PET images using BAPL score between the PET_20m_ and sPET_20m_ images.

	PET_20m_ (GT)											
	Reader 1				Reader 2				Reader 3			
	BS1	BS2	BS3	Total	BS1	BS2	BS3	Total	BS1	BS2	BS3	Total
**sPET**_**20m**_												
sBS1	13	2	0	15	15	5	0	20	12	4	0	16
sBS2	3	16	0	19	1	13	0	14	4	14	0	18
sBS3	0	0	24	24	0	0	24	24	0	0	24	24
Total	16	18	24	58	16	18	24	58	16	18	24	58

*BS* BAPL score of ground truth, *GT* ground truth, *sBS* BAPL score of the synthetic PET image.

We evaluated the intra-observer agreement using Cohen’s weighted kappa by comparing the BAPL scores between the sPET_20m_ and PET_20m_ images. Clinicians’ Cohen’s weighted kappa was 0.902 (DY), 0.887 (YJ), and 0.844 (JE), with a mean value of 0.878.

## Discussion

In this study, we investigated the feasibility of a deep-learning-based reconstruction approach using short-time acquisition PET scans. We used PET images acquired for 2 and 20 min as input and target images, respectively. Quantitative and qualitative analyses showed that the proposed method produces efficient synthetic PET images from short-scanning PET images. We calculated image-quality metrics (such as PSNR, SSIM, and NRMSE) for model evaluation between the synthetic images and ground-truth images (standard scanning images). Overall, the proposed method improved the image quality by suppressing the noise in short-scanning images. Note that the SSIM index depends on the parameters (${K}_{1}$ and ${K}_{2}$). In our study, the average SSIM index for the synthetic images increased from 0.8818 to 0.9939 when ${K}_{1}, {K}_{2}$ increased from $0.0002\,\mathrm{ to }\,0.0007$ and $0.01\, \mathrm{to }\,0.03$, respectively. However, in this case, the differences in the SSIM index were very small. Our deep-learning method also improved the image qualities of the 5-min images of the ADNI dataset, even though the test domain significantly differs from our training domain.

We adopted the GAN framework with an additional mean-squared loss between the synthetic sPET_20m_ image and the PET_2m_ image. The performance of the proposed network was compared with that of the conventional U-net. The U-net minimizes only the pixelwise loss between the synthetic PET image and ground-truth (i.e., PET_20m_) image, resulting in an over-smoothed image, whereas the proposed approach clearly reconstructs the detailed structures of the brain (Fig. 2)^25^. In terms of quality measurements, such as PSNR, NRMSE, and SSIM, the proposed method outperformed the U-net. The time taken to generate a synthetic single sPET_20m_ image from a PET_2m_ image was within a few milliseconds on the GPU system, which would make the proposed method adequate for clinical use.

Some previous studies have also tried to reduce noise and improve image quality using a deep-learning technique in PET imaging^5–9^. Most of these studies aimed at maintaining the quality of the PET image while reducing the injection dose of radiopharmaceuticals in order to minimize radiation exposure. They showed that the image quality of low-dose PET could be restored like the original PET images obtained with standard protocols while reducing the conventional radiopharmaceutical dose by up to 99%. However, they all used synthesized low-dose data (i.e., a small amount of data selected from the entire acquisition period), which may differ from the measured data obtained from the true low dose. A feasibility study on real data is needed for clinical use. One study restored a low-quality PET image taken in 3 min to match a standard image taken in 12 min^7^. This study differs from ours in that it used MRI information taken together to restore image quality. Considering the absence of a PET/MRI scanner in most hospitals, the proposed method using PET images only could be used in general clinical practice. Another study reported that using a 5-min PET image, one frame of 20-min data without deep-learning methods, did not relevantly affect the accuracy of disease discrimination^26^. The advantage of our method is that it can generate PET images like those of full-time scanning images with only 2-min data in any part, regardless of the frame. In our study, no comparison of diagnostic accuracy between PET images obtained by our method and 5-min PET images was done. However, if PET image reconstruction with short-time data is required, we think that our method, along with the PET imaging method using one frame 5-min data, can broaden the range of options that can be selected according to the situation.

Since amyloid PET images are used in hospitals to care for patients with memory impairment, deep-learning-generated images must have an image quality similar enough to the original image that it can be used for interpretation in the clinics. In this study, we used several methods to decide whether generated images could be available clinically. We did tests to find an answer to the following questions: Can physicians distinguish between PET_20m_ and generated sPET_20m_ images? What is the difference in visual interpretation results? What is the difference between quantitative analysis using SUVR in both images?

When PET_20m_ and generated sPET_20m_ images were presented at the same time to three nuclear medicine physicians who were in charge of clinical reading, the accuracy of the selection of the PET_20m_ images was within 40–60%. This suggests that synthetic PET images generated by our method are almost indistinguishable from the real PET image. Next, we did the BAPL scoring test to assess the intra-observer agreement and diagnostic accuracy. In our study, Cohen's weighted kappa was above 0.84, which indicated an almost perfect intra-observer agreement. We also did BAPL scoring on generated PET images, which we compared with the ground-truth scores. In the strong positive cases (BAPL 3), all three physicians showed a 100% accuracy, but in the negative (BAPL 1) and weak positive (BAPL 2) cases, between 5 and 8 of the 58 patients were false-positive or false-negative. It is already known that the amyloid PET study itself, even if obtained according to a conventional protocol, can cause misclassification when visually read. Some studies have reported that about 10% of the results may be inconsistent^27, 28^. In addition, some errors from the deep-learning algorithm could be added, so we think that the misclassification has increased a little in our study. We also think that the physician’s opinion may have some influence on the interpretation of how much the amyloid uptake is positive in the visual reading that distinguishes BAPL 1 and 2. Few studies have evaluated the accuracy of physicians’ interpretations among studies related to deep learning on a subject similar to ours. One study showed 89% accuracy when read using deep-learning-generated PET images, which is very similar to our result^6^.

In order to make up for the weak points of the visual reading, SUVR is used as a quantitative indicator in routine practice to infer the severity or prognosis of the disease^23^. In the generated brain PET images of this study, regional SUVRs were not significantly different from the values of ground-truth images in negative and positive cases (*p* > 0.05). In the Bland–Altman analysis, the mean of the difference was 0.005 in the negative case and 0.024 in the positive case, and the limits of agreement of each region were small. That is, our deep-learning model can generate images with SUVR values that are comparable to those of the original PET images. We obtained similar results by means of temporal and external validations, which allowed us to reconfirm this fact. Taken together, these results suggest that the synthetic amyloid PET images generated by our deep-learning method could be used for clinical reading purposes.

Our study has some limitations that need to be considered for clinical use. First, our deep-learning model trained on FBB PET with 2-min data should be tested under various acquisition conditions. Using multicenter datasets for training or incorporating domain adaptation techniques could improve image quality, which is a part of our future work^29, 30^. In this study, in order to avoid overfitting, we evaluated our model using the ADNI data, a completely different dataset, and our hospital data obtained at a different time from the training dataset. Second, in our study, we empirically chose 2-min images as a training dataset for short scanning. However, 2-min PET images may not be optimal. More rigorous analysis may be needed to choose the proper short-scanning image. Third, we generated only trans-axial PET images in this study. Although interpretation guidelines for FBB PET recommend using trans-axial PET images for clinical reading, coronal and sagittal PET images have also been used recently for reading. In the next study, we need to apply our deep-learning model to generate three orthogonal PET images. In addition, the application of a 3-dimensional model and finding the optimal hyperparameters is a problem to be solved in the future.

In conclusion, we presented an image-restoration method using a deep-learning technique to yield a clinically acceptable amyloid brain PET image with short-time data. Qualitative and quantitative analysis by means of internal, temporal, and external validations showed that the image quality and quantitative value of the generated PET images were very similar to those of the original images. Although more evaluation and validation are needed, we found that applying deep-learning techniques to amyloid brain PET images can reduce acquisition time and provide clinically equivalent interpretable images as standard images.

## Supplementary Information

Supplementary Informations.

## Data Availability

The datasets generated during and/or analyzed during the current study are available from the corresponding author on reasonable request.

## References

[1] Mallik A, Drzezga A, Minoshima S (2017). Clinical amyloid imaging. Semin. Nucl. Med..

[2] Minoshima S (2016). SNMMI procedure standard/EANM practice guideline for amyloid PET imaging of the brain 1.0. J. Nucl. Med..

[3] Duffy IR, Boyle AJ, Vasdev N (2019). Improving PET imaging acquisition and analysis with machine learning: a narrative review with focus on Alzheimer's disease and oncology. Mol. Imaging.

[4] Zhu G (2019). Applications of deep learning to neuro-imaging techniques. Front. Neurol..

[5] Gatidis S (2016). Towards tracer dose reduction in PET studies: simulation of dose reduction by retrospective randomized undersampling of list-mode data. Hell. J. Nucl. Med..

[6] Chen KT (2019). Ultra-low-dose ^18^F-florbetaben amyloid PET imaging using deep learning with multi-contrast MRI inputs. Radiology.

[7] Xiang L (2017). Deep auto-context convolutional neural networks for standard-dose PET image estimation from low-dose PET/MRI. Neurocomputing.

[8] Ouyang J (2019). Ultra-low-dose PET reconstruction using generative adversarial network with feature matching and task-specific perceptual loss. Med. Phys..

[9] Gong K, Guan J, Liu C, Qi J (2019). PET image denoising using a deep neural network through fine tuning. IEEE Trans. Radiat. Plasma Med. Sci..

[10] Kang E, Min J, Ye JC (2017). A deep convolutional neural network using directional wavelets for low-dose X-ray CT reconstruction. Med. Phys..

[11] Chen H (2017). Low-dose CT denoising via convolutional neural network. Biomed. Opt. Express.

[12] Jeong YJ, Yoon HJ, Kang DY (2017). Assessment of change in glucose metabolism in white matter of amyloid-positive patients with Alzheimer disease using F-18 FDG PET. Medicine.

[13] Goodfellow, I. *et al*. Generative adversarial nets. in *NIPS* 2014 (2014).

[14] Wolterink JM, Leiner T, Viergever MA, Isgum I (2017). Generative adversarial networks for noise reduction in low-dose CT. IEEE Trans. Med. Imaging..

[15] Park HS (2019). Unpaired image denoising using a generative adversarial network in X-ray CT. IEEE Access..

[16] Ye JC, Han Y, Cha E (2017). Deep convolutional framelets: a general deep learning framework for inverse problems. SIAM J. Imaging Sci..

[17] Ioffe, S. & Szegedy, C. Batch normalization: accelerating deep network training by reducing internal covariate shift. Preprint at arXiv:1502.03167 (2017).

[18] Nair, V. & Hinton, G.E. Rectified linear units improve restricted Boltzmann machines. in *ICML.* 807–814 (2010).

[19] Chui CK (2014). An Introduction to Wavelets.

[20] Ronneberger, O., Fischer, P. & Brox, T. U-Net: convolutional networks for biomedical image segmentation. in *MICCAI* 2015 (2015).

[21] Gu J (2018). Recent advances in convolutional neural networks. Pattern Recogn..

[22] Zhang YC, Kagen AC (2017). Machine learning interface for medical image analysis. J. Digit. Imaging.

[23] Bullich S (2017). Optimized classification of ^18^F-Florbetaben PET scans as positive and negative using an SUVR quantitative approach and comparison to visual assessment. Neuroimage Clin..

[24] Barthel H, Sabri O (2011). Florbetaben to trace amyloid-β in the Alzheimer brain by means of PET. J. Alzheimers Dis..

[25] Johnson, J., Alahi, A. & Fei-Fei, L. Perceptual Losses for Real-Time Style Transfer and Super-Resolution. in *ECCV* 2016 (2016).

[26] Tiepolt S (2013). Influence of scan duration on the accuracy of β-amyloid PET with florbetaben in patients with Alzheimer’s disease and healthy volunteers. Eur. J. Nucl. Med. Mol. Imaging..

[27] Oh M (2018). Clinical significance of visually equivocal amyloid PET findings from the Alzheimer's disease neuroimaging initiative cohort. NeuroReport.

[28] Yamane T (2017). Inter-rater variability of visual interpretation and comparison with quantitative evaluation of 11C-PiB PET amyloid images of the Japanese Alzheimer’s Disease Neuroimaging Initiative (J-ADNI) multicenter study. Eur. J. Nucl. Med. Mol. Imaging.

[29] Gao Y, Li Y, Ma K, Zheng Y (2019). A universal intensity standardization method based on a many-to-one weak-paired cycle generative adversarial network for magnetic resonance images. IEEE Trans. Med. Imaging..

[30] Chen, J. *et al*. Generative adversarial networks for video-to-video domain adaptation. in *AAAI* (2020).

