# Supplementary material for: Restoration of amyloid PET images obtained with short-time data using a generative adversarial networks framework
Source: Sci Rep. 2021 Mar 1;11:4825. doi: 10.1038/s41598-021-84358-8 (PMC7921674; doi:10.1038/s41598-021-84358-8)
Supplement: Supplementary file 1 — Supplementary Informations. [file 41598_2021_84358_MOESM1_ESM.docx]

**Supplementary information**

**Restoration of amyloid PET images obtained with short-time data using a generative adversarial networks framework**

Young Jin Jeong, M.D.^1, 2^, Hyoung Suk Park, Ph.D.^4^, Ji Eun Jeong, M.D.^1^, Hyun Jin Yoon, Ph.D.^1^, Kiwan Jeon, Ph.D.^4^, Kook Cho, Ph.D.^5^, Do-Young Kang, M.D., Ph.D.^1, 2, 3*^

^1^Department of Nuclear Medicine, Dong-A University Hospital, Dong-A University College of Medicine, Busan, Republic of Korea.

^2^Institute of Convergence Bio-Health, Dong-A University, Busan, Republic of Korea.

^3^Department of Translational Biomedical Sciences, Dong-A University, Busan, Republic of Korea.

^4^National Institute for Mathematical Science, Daejeon, Republic of Korea.

^5^College of General Education, Dong-A University, Busan, Republic of Korea.

**These authors contributed equally to this work: Young Jin Jeong and Hyoung Suk Park**

**Corresponding Author:**

Do-Young Kang, M.D., Ph.D.

Department of Nuclear Medicine, Dong-A University College of Medicine and Medical Center

1, 3ga, Dongdaesin-dong, Seo-gu, Busan, 602-715, South Korea

Tel: 82-51-240-5630, Fax: 82-51-242-7237, E-mail: dykang@dau.ac.kr

| Temporal validation set | Positive group | | | Negative group | | |
| --- | --- | --- | --- | --- | --- | --- |
|  | s20min | 20min | p-value^*^ | s20min | 20min | p-value^†^ |
| Frontal lobe | 1.50 ± 0.32 | 1.48 ± 0.34 | 0.621 | 1.22 ± 0.17 | 1.20 ± 0.17 | 0.635 |
| Parietal lobe | 1.58 ± 0.29 | 1.55 ± 0.30 | 0.684 | 1.28 ± 0.16 | 1.29 ± 0.19 | 0.899 |
| Temporal lobe | 1.40 ± 0.21 | 1.37 ± 0.22 | 0.546 | 1.22 ± 0.16 | 1.21 ± 0.16 | 0.975 |
| Occipital lobe | 1.59 ± 0.27 | 1.56 ± 0.29 | 0.621 | 1.39 ± 0.19 | 1.39 ± 0.23 | 0.874 |
| Striatum | 1.28 ± 0.27 | 1.26 ± 0.30 | 0.701 | 1.29 ± 0.26 | 1.29 ± 0.28 | 0.825 |
| Global brain | 1.46 ± 0.25 | 1.43 ± 0.26 | 0.717 | 1.24 ± 0.19 | 1.25 ± 0.17 | 0.899 |

**Supplementary Table 1.** Comparison of SUVRs in the representative areas between the positive and negative groups in the internal validation dataset. There is no statistical difference between s20min and 20min PET images in the representative areas. Values are presented as mean ± SD (standard deviation). ^*^: independent t-test; ^†^: Mann-Whitney U test


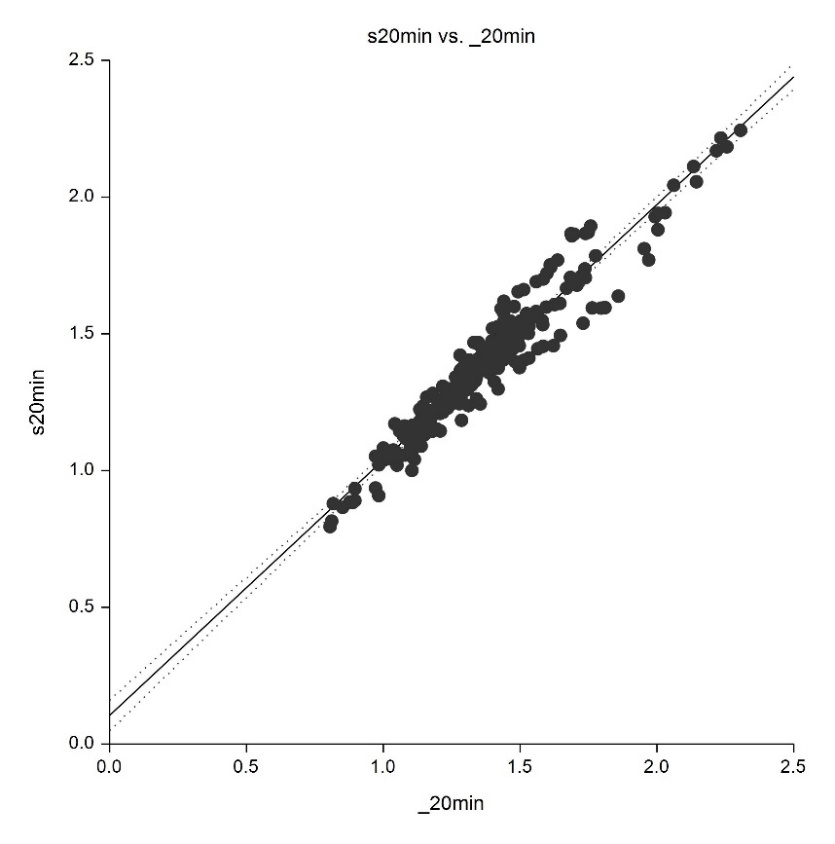


**Supplementary Figure 1.** Correlation analysis of the whole representative areas shows a trend of a strong positive relationship of SUVR in the internal validation dataset (r = 0.964, p < 0.001)

| Temporal validation set | Positive group | | | Negative group | | |
| --- | --- | --- | --- | --- | --- | --- |
|  | s20min | 20min | p-value^*^ | s20min | 20min | p-value^*^ |
| Frontal lobe | 1.71 ± 0.22 | 1.72 ± 0.21 | 0.818 | 1.48 ± 0.15 | 1.48 ± 0.17 | 0.908 |
| Parietal lobe | 1.88 ± 0.24 | 1.86 ± 0.23 | 0.963 | 1.64 ± 0.12 | 1.63 ± 0.13 | 0.862 |
| Temporal lobe | 1.43 ± 0.18 | 1.42 ± 0.18 | 0.963 | 1.25 ± 0.14 | 1.23 ± 0.14 | 0.729 |
| Occipital lobe | 1.68 ± 0.20 | 1.67 ± 0.20 | 0.890 | 1.45 ± 0.11 | 1.43 ± 0.11 | 0.564 |
| Striatum | 1.54 ± 0.21 | 1.51 ± 0.22 | 0.613 | 1.26 ± 0.15 | 1.22 ± 0.16 | 0.326 |
| Global brain | 1.65 ± 0.20 | 1.64 ± 0.19 | 0.854 | 1.42 ± 0.12 | 1.40 ± 0.13 | 0.644 |

**Supplementary Table 2.** Comparison of SUVRs in the representative areas between the positive and negative groups in the temporal validation dataset. There is no statistical difference between s20min and 20min PET images in the representative areas. Values are presented as mean ± SD. ^*^: Mann-Whitney U test


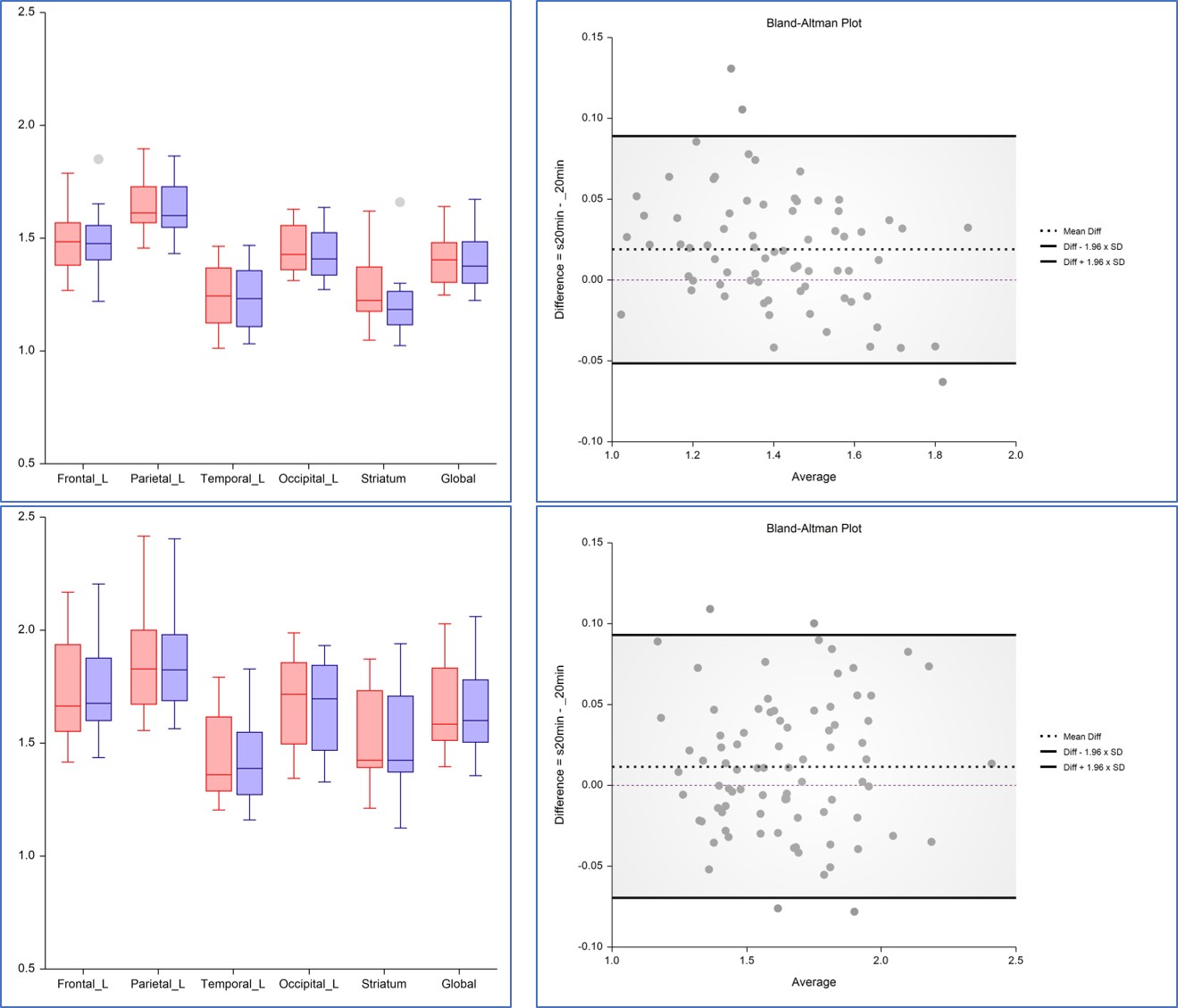


**Supplementary Figure 2.** Barplots (left column) and Bland-Altman analysis (right column) show the difference of SUVR between the positive (lower row) and negative (upper row) groups in the temporal validation dataset. In Bland-Altman analysis, the mean difference is 0.012 (lower limit, -0.070; upper limit, 0.093) in the positive group and 0.019 (lower limit, -0.051; upper limit, 0.089) in the negative group.

| External validation set | Positive group | | | Negative group | | |
| --- | --- | --- | --- | --- | --- | --- |
|  | s20min | 20min | p-value^*^ | s20min | 20min | p-value^*^ |
| Frontal lobe | 1.64 ± 0.22 | 1.68 ± 0.24 | 0.447 | 1.45 ± 0.08 | 1.49 ± 0.08 | 0.155 |
| Parietal lobe | 1.76 ± 0.20 | 1.82 ± 0.21 | 0.260 | 1.57 ± 0.11 | 1.63 ± 0.02 | 0.077 |
| Temporal lobe | 1.42 ± 0.18 | 1.45 ± 0.20 | 0.613 | 1.29 ± 0.06 | 1.30 ± 0.06 | 0.621 |
| Occipital lobe | 1.61 ± 0.19 | 1.65 ± 0.20 | 0.497 | 1.44 ± 0.06 | 1.48 ± 0.07 | 0.094 |
| Striatum | 1.45 ± 0.25 | 1.48 ± 0.28 | 0.633 | 1.23 ± 0.11 | 1.24 ± 0.11 | 0.809 |
| Global brain | 1.58 ± 0.20 | 1.61 ± 0.21 | 0.458 | 1.40 ± 0.08 | 1.43 ± 0.08 | 0.216 |

**Supplementary Table 3.** Comparison of SUVRs in the representative areas between the positive and negative groups in the external validation dataset. There is no statistical difference between s20min and 20min PET images in the representative areas. Values are presented as mean ± SD. ^*^: independent t-test


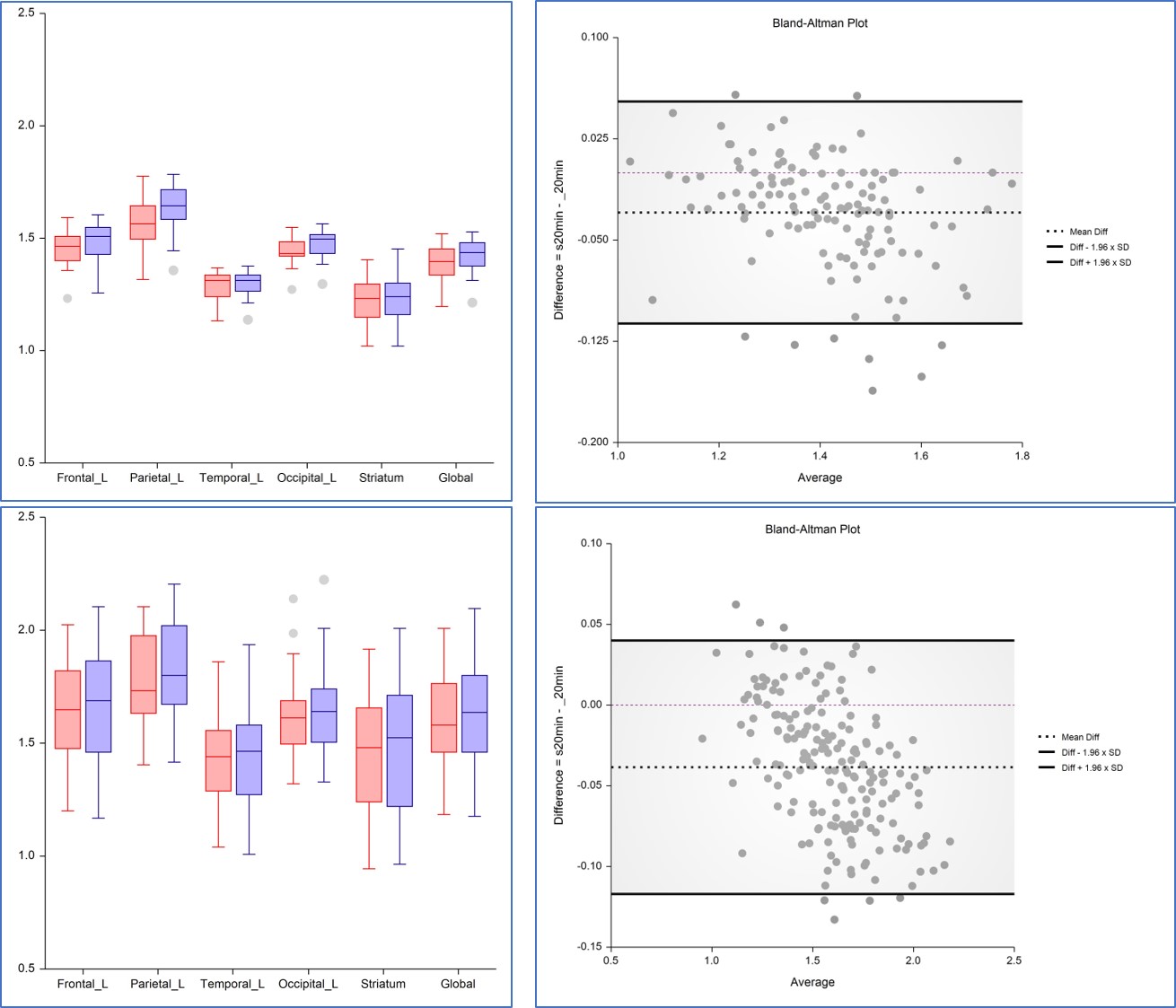


**Supplementary Figure 3.** Barplots (left column) and Bland-Altman analysis (right column) show the difference of SUVR between the positive (lower row) and negative (upper row) groups in the external validation dataset. In Bland-Altman analysis, the mean difference is -0.039 (lower limit: -0.117, upper limit: 0.040) in the positive group and -0.029 (lower limit: -0.112, upper limit: 0.053) in the negative group.
